# mirTarPri: Improved Prioritization of MicroRNA Targets through Incorporation of Functional Genomics Data

**DOI:** 10.1371/journal.pone.0053685

**Published:** 2013-01-09

**Authors:** Peng Wang, Shangwei Ning, Qianghu Wang, Ronghong Li, Jingrun Ye, Zuxianglan Zhao, Yan Li, Teng Huang, Xia Li

**Affiliations:** College of Bioinformatics Science and Technology, Harbin Medical University, Harbin, China; National Central University, Taiwan

## Abstract

MicroRNAs (miRNAs) are a class of small (19–25 nt) non-coding RNAs. This important class of gene regulator downregulates gene expression through sequence-specific binding to the 3′untranslated regions (3′UTRs) of target mRNAs. Several computational target prediction approaches have been developed for predicting miRNA targets. However, the predicted target lists often have high false positive rates. To construct a workable target list for subsequent experimental studies, we need novel approaches to properly rank the candidate targets from traditional methods. We performed a systematic analysis of experimentally validated miRNA targets using functional genomics data, and found significant functional associations between genes that were targeted by the same miRNA. Based on this finding, we developed a miRNA target prioritization method named mirTarPri to rank the predicted target lists from commonly used target prediction methods. Leave-one-out cross validation has proved to be successful in identifying known targets, achieving an AUC score up to 0. 84. Validation in high-throughput data proved that mirTarPri was an unbiased method. Applying mirTarPri to prioritize results of six commonly used target prediction methods allowed us to find more positive targets at the top of the prioritized candidate list. In comparison with other methods, mirTarPri had an outstanding performance in gold standard and CLIP data. mirTarPri was a valuable method to improve the efficacy of current miRNA target prediction methods. We have also developed a web-based server for implementing mirTarPri method, which is freely accessible at http://bioinfo.hrbmu.edu.cn/mirTarPri.

## Introduction

MicroRNAs (miRNAs) are a class of small (19–25 nt) non-coding RNAs that reduce the abundance and translational efficiency of mRNAs. These non-coding RNAs play a major role in human regulatory networks and diverse biological phenomena [1]–[3]. Information about miRNA targets can be used for the study of complex RNA regulatory networks, disease diagnosis and pharmacogenomics [4]–[6]. Because of the absence of a high-throughput model for specific miRNA target recognition, better methods for the identification of miRNA targets are urgently needed. Several computational target prediction approaches, such as TargetScan, PicTar, miRanda, PITA, DIANA-microT and RNAhybrid, have been developed to predict target genes [7]–[13]. These methods are mostly based on characteristics of miRNA seed region such as sequence matches, G-U wobble and thermodynamic duplex stability. Although the seed region is evolutionarily conserved, it is not reliable by itself to identify miRNA targets. It has been shown that approximately 70% of predictions are false positive targets [11], [14]. Identification of true positive targets from the large predicted target lists is complex, expensive and laborious [15]. Therefore, novel approaches for prioritizing target lists from traditional prediction methods are needed to construct a workable target list for subsequent experimental studies.

Several machine-learning-based classification methods have been developed to improve the accuracy of miRNA target prediction, such as TargetBoost [16] and miTarget [17]. A previous study has shown that miTarget didn’t consider conservation information in order to avoid a loss of sensitivity; however, as a consequence, the number of false positive targets remains high [18], [19]. Moreover, because of a lack of negative controls, current machine learning approaches rely on artificially generated negative examples for training purposes, which also results in a high false positive rate [20]. In addition, several target prediction methods that incorporated expression data have been developed [21]–[24]. However, there are a certain number of documented miRNAs that suppress the translational activities of the target mRNA. In this case, there is no direct effect on the expression level of the target mRNA; thus, these type of targeting pairs cannot be observed in gene expression profiles [25], [26].

Some observed phenotypes are likely to be caused by complex regulation of several targets regulated by a single miRNA [27], [28]. To further understand the regulatory mechanisms of miRNAs in complex cellular systems, functional associations have been identified between target genes based on accumulated functional genomics data sets [29], [30]. Several studies have revealed that miRNA targets were often involved in highly correlated functional modules (i.e., they shared similar biological functions or were close to each other in protein-protein interaction (PPI) networks) [31]–[33]. These target genes are often regulated simultaneously and share the same expression patterns [34]–[36].

In previous work, we have prioritized human cancer miRNAs based on genes’ functional consistency [37]. In this study, we developed a miRNA target prioritization method named mirTarPri that used functional genomics data to rank predicted target lists. Leave-one-out cross validation has proved to be successful in identifying 1,799 validated miRNA-target pairs with an AUC score up to 0.84. Validation of microarray and pulse-labeing SILAC data has proved that mirTarPri was an unbiased method. Applying mirTarPri to prioritize the results of commonly used target prediction databases, including TargetScan, PicTar, miRanda, PITA, DIANA-microT and RNAhybrid allowed us to find more positive targets. We have made mirTarPri available on a web-based server, and a full list of prioritized miRNA target lists from the six prediction databases is freely accessible at http://bioinfo.hrbmu.edu.cn/mirTarPri.

## Materials and Methods

### Validated and Predicted miRNA Target Dataset

We downloaded lists of human miRNAs and their associated targets from three high-quality online miRNA reference databases: TarBase (v.5c) [38], miR2Disease (version Jan, 2010) [39] and miRecord (version Nov, 2010) [40]. These databases store manually curated collections of experimentally supported miRNA targets. After combining these databases, 1,799 miRNA-target pairs were collected in our study. The predicted miRNA targets were downloaded from commonly used prediction databases: TargetScan (version 5.1), PicTar (version Mar, 2007), miRanda (version Nov, 2010), PITA (version 6.0) and DIANA-microT (version 3.0). Since RNAhybrid did not provide predicted results, we used RNAhybrid (version 2.1) software to predict miRNA target sites on human transcripts using default parameters. The human transcript sequences were downloaded from Ensembl (GRCH37) [41].

### Gene Ontology Dataset

Gene Ontology (GO) comprises three orthogonal ontologies, biological processes (BP), molecular functions (MF) and cellular components (CC), which provide a controlled vocabulary for describing genes or their encoded products with predefined terms [42]. GO terms and their relationships are represented in the form of a Directed Acyclic Graph (DAG). We downloaded the gene-annotation dataset for Human (version May, 2011), Arabidopsis thaliana (version Feb, 2012) and Mouse (version Apr, 2012) from the official GO website.

### PPI Network Datasets

We downloaded PPI data from six databases: HPRD (release 9.0) [43], BIND (release 1.0) [44], MINT (version 2.5) [45], BioGrid (version 3.1.90 ) [46], IntAct (version 2.0) [47] and OPHID (version 1.95) [48]. OPHID is an integrated network that contains data from the other five datasets. Self-loops of one protein and round-trips between two proteins were refined to one interaction.

### Measurement of Functional Similarity and Network Closeness

A basic and critical step in our method was to measure the functional associations between miRNA targets. For each target-gene pair, mirTarPri measured their associations in two ways: functional similarity based on GO annotations [49] and network closeness based on PPI networks [29].

Semantic similarity was used to assess the degree of relatedness between two words or entities in taxonomy. It could be alternatively evaluated based on the notion of information content [50]. When biological entities were described using a common schema, such as an ontology, semantic similarity could be used as a measure to compare them by means of their annotations. GO is well organized and structured as DAG corresponding to orthogonal categories. Nodes in the graph represent terms that describe gene product function. Previous studies have demonstrated that semantic similarity base on GO annotations could be used to quantify the functional similarity between gene products [49], [51], [52]. Here we used the theory of information content (IC) to define semantic similarity measure from Resnik. The similarity of two terms was calculated that they shared common information in an ontology represented as a DAG, which was always indicated by the specific common ancestor. The use of IC was a reliable way to measure how specific and informative a term was. The IC value of a term, 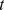, could be calculated as the negative log likelihood:
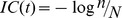
Where 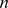 is the number of genes mapped to term 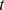, and 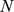 is the total number of genes in the whole human genome. Quantifying IC in this way makes intuitive sense; as the IC value increases, the term function becomes more specific [50]. The functional similarity (FS) score between two target genes, 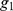 and 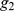, was previously defined and used as the IC value of the most informative common ancestor among the terms mapped by 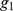 and those by 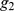 [52], [53], as shown in the following equation:



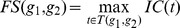
Here, 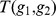 denotes the set of all common ancestor terms mapped by 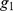 and 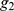. A higher FS score indicates that two genes share more information in common and are more similar. The average functional similarity (AFS) score between a candidate target, 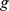 and a group of 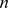 experimentally validated targets, 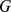, was defined as follows:



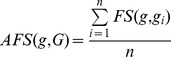
Where 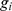 is a member of group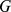.

Network closeness (NC) score of two target genes 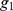 and 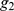 was defined as reciprocal of shortest distance (DIS) between gene products nodes on network using Dijkstra’s algorithm:
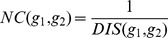



The average network closeness (ANC) score between a candidate target, 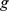, and a group of 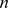 experimentally validated targets, 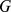, was defined as follows:
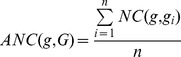
Where 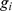 is a member of group 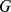.

### Multiple Data Rank Fusion

We combined ranks from separate functional genomics data using the following 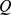 statistic formula, which was implemented and used in a previous multiple rank fusion study [54]:
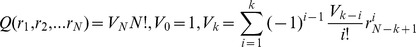
where 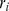 is the rank ratio for data source 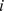, 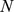 is the number of data sources used, and 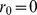. The time complexity of this formula is 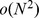.

### Enrichment Score

To measure the performance of mirTarPri for prioritizing commonly used miRNA target predictions, we used an enrichment-fold method to quantify the efficiency of mirTarPri. We used the enrichment-fold score (ES) defined as 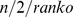 [55] for a ranked list of n genes. For instance, if mirTarPri gave the highest rank to a known target gene that was ranked first in a list of 100 genes by the target prediction databases, the enrichment score was 50. If the gene was given a rank of 100, the enrichment score was 0.5. An enrichment score of 1 indicated a middle rank.

### Random Gene Set

In the analysis of functional similarity and network closeness between validated targets, we used random gene sets as controls. If a miRNA had 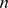 experimentally validated targets, we randomly selected 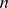 genes from the whole human genome. Then, the FS score and NC score were calculated for each gene pair in the randomly selected group. For each miRNA, 1000 random groups were generated. In each leave-one-out cross validation, 99 genes were also randomly selected from the whole human genome.

### Principle of mirTarPri

**Figure 1:**
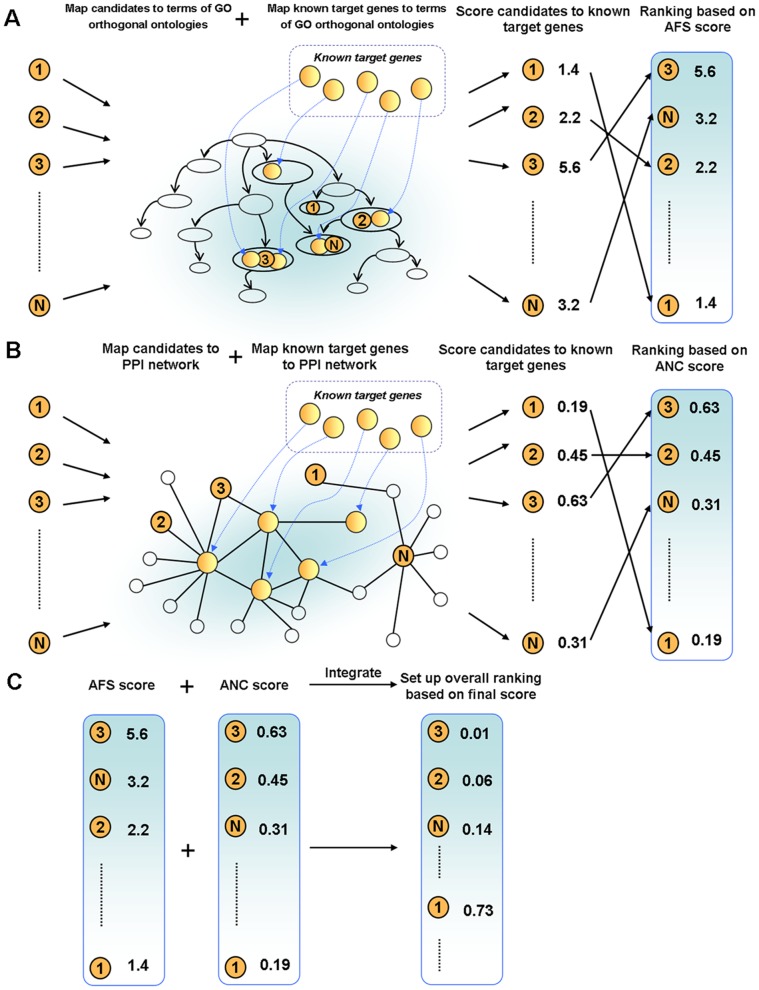
Conceptual schematic of the mirTarPri procedure. First, each candidate target gene is mapped to GO terms from orthogonal ontologies to measure its AFS score relative to known targets. The candidate targets are then ranked according to their AFS scores. Second, each candidate target gene is mapped to the PPI network to measure an ANC score relative to known targets. The candidate targets are then ranked according to their ANC scores. Finally, the two rankings based on the AFS and ANC scores are combined into a single rank using the Q statistic method.

We hypothesized that the positive functional associations between genes that were targeted by the same miRNA could be quantified and used to improve the prioritization of miRNA target prediction results. In this study, we proposed a method named mirTarPri. Using this method, candidate targets that are prioritized for a specific miRNA according to semantic similarity and proximity to experimentally validated targets. There are three major steps to prioritize using mirTarPri. The first step (Figure 1A) maps the experimentally validated and candidate targets of each miRNA to GO terms from one of three orthogonal ontologies. For a candidate target, the AFS score is calculated for this candidate and the experimentally validated group. The candidate target list is ranked according to its AFS score. In the second step (Figure 1B), experimentally validated and candidate targets for each miRNA are mapped to the PPI network. Then the ANC score is calculated for each candidate and rank them accordingly. In the third step (Figure 1C), the two ranks based on the AFS and ANC scores are combined for each candidate target into a single rank using multiple rank fusion method. For each rank, the Q statistic method generates an integrated score. This rank indicates the overall priority for each candidate target list.

## Results

### Systematic Analysis of Functional Similarity between Experimental Validated miRNA Targets

**Figure 2:**
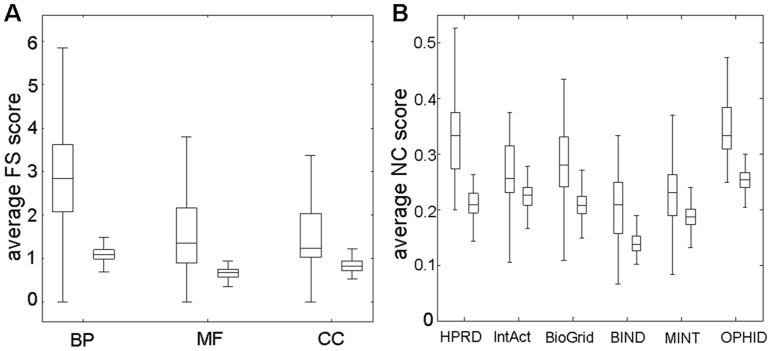
Systematic analysis of functional similarity and network closeness between experimental validated miRNA targets. (A) Comparison of average FS score between experimentally validated targets (left box of each pair, representing average FS score for every miRNA-target gene pair) and randomly generated genes (right box of each pair) targeted by each miRNA based on three orthogonal ontologies of GO. (B) Comparison of average NC score between experimentally validated targets (left box of each pair) and randomly generated targets (right box of each pair) based on six PPI networks.

Previous studies have revealed that miRNA target genes in the same module shared similar GO annotations [32]. It has also been reported that miRNAs had different propensities to target genes involved in different biological processes or functional categories [11], [25], [31]. For example, the miR-17-18-19-20 gene cluster was involved in solid tumors [56]. Genes targeted by this miRNA cluster overwhelmingly played important roles in growth control, including both oncogenes and genes that repressed growth [11]. To determine whether these functional associations could be used as a scoring method, targets were mapped to three orthogonal gene ontologies (BP, MF, or CC). For each pair of target genes from each group (i.e., targeted by the same miRNA), we calculated the FS score (see Materials and Methods). The FS score indicated functional similarities between two gene products by combining the semantic similarities of their associated terms [49]. We found a high level of functional similarity between target genes for each orthogonal ontology. The average FS score of each gene pair targeted by the same miRNA was significantly higher than those of randomly generated gene pairs (Figure 2A). For each miRNA targeting 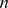 genes, we randomly generated a set of 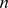 genes as simulated targets. The fold-change value and significance level of the average FS score generated using BP terms exceeded those for MF and CC (Table 1). BP represents a sophisticated functional ontology containing more than 8,000 terms, which is approximately 2.5 and 7.6 times greater than the MF (approximately 3,000 terms) and CC (approximately 1,000 terms) ontologies, respectively. The AFS score between each miRNA target and simulated gene were compared using a Mann-Whitney U-test, and the P-values for the three orthogonal ontologies were statistically significant.

**Table 1:** Functional similarity analysis of miRNA targets based on GO.

	BP	CC	MF
Number of terms	8373	1089	3398
Number of genes	14623	16418	15571
Mean FS score betweenvalidated targets	2.9069	1.4978	1.6503
Mean FS score betweenrandom genes	1.1086	0.8382	0.6837
Fold change	2.6221	1.7869	1.4138
U-test P value	2.38e-35	1.50e-23	3.81e-22

**Figure 3:**
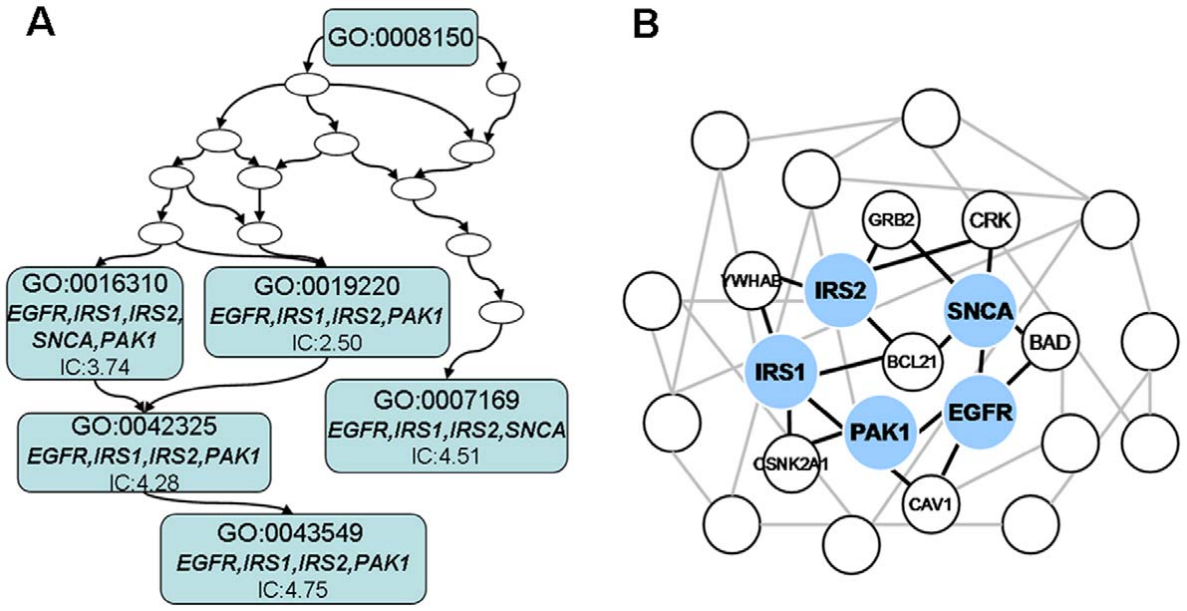
An illustration of functional similarity between genes targeted by the same miRNA. Five target genes (*PAK1*, *SNCA*, *EGFR*, *IRS1* and *IRS2*) for hsa-miR-7 were mapped to BP terms (A) and the HPRD network (B). Five targets were mapped to the common term GO: 0016310 with a significantly higher IC than that of random genes (*p-value*<0.01). Five target gene products (grey) were close to each other on the HPRD network, and the average NC score was 0.59.

For example, experimentally validated targets of hsa-miR-7 (*EGFR*, *IRS1*, *IRS2*, *SNCA* and *PAK1*) were mapped to phosphorylation-related BP terms (Figure 3A). All five targets were mapped to GO: 0016310 (phosphorylation, IC = 3.74), and four of the five targets were mapped to GO: 0043549 (regulation of kinase, IC = 4.75). These results indicated that experimentally validated targets of hsa-miR-7 shared similar biological functions. The same tendencies were observed on MF and CC (Figure S1). In addition, three of the five targets were mapped to GO: 0004672 (protein kinase activity, IC = 8.55) on MF, and four of the five targets were mapped to GO: 0005829 (cytosol, IC = 3.24) on CC.

### Systematic Analysis of Network Closeness between Experimental Validated miRNA Targets

To evaluate whether miRNA targets are close to each other in PPI networks, we treated the targets as nodes within a large undirected graph and considered the network closeness of these nodes. Previous studies based on human PPI networks have shown that the genes encoding two interacting proteins tended to be under similar miRNA regulation [33] and to have similar mRNA expression profiles [34]–[36]. For every two target genes of each group, we calculated NC score between them within the network. Compared with simulated genes, the target genes in the network occupied a narrow niche. The average NC score between experimentally validated target genes was significantly higher (*p-value*<1.0e-10) than that of simulated genes (Table 2). In comparing six PPI networks, we found that the performance of HPRD was higher than the others with 1.6-fold change (Mann-Whitney U-test, *p-value*<3.80e-41, Figure 2B). For this analysis, we used the same set of random genes mentioned above in the GO functional similarity analysis; the P-values of all six networks were statistically significant.

**Table 2:** Analysis of the functional similarities of miRNA targets based on six PPI networks.

	HPRD	BioGrid	IntAct	MINT	BIND	OPHID
Interactions	39189	51355	39794	14342	9670	69495
Nodes	9465	10030	7752	5208	3309	11377
Annotated targets	1385	1362	1205	926	738	1541
Mean NC score between validated targets	0.34	0.28	0.28	0.24	0.21	0.36
Mean NC score between random targets	0.21	0.21	0.22	0.19	0.14	0.25
Fold change	1.62	1.33	1.27	1.26	1.5	1.44
U-test P value	3.80e-41	3.05e-12	1.40e-18	2.18e-14	8.14e-10	1.07e-37

Using PPI network, we found that the validated targets (*EGFR*, *IRS1*, *IRS2*, *SNCA* and *PAK1*) of hsa-miR-7 were close to each other. In HPRD, the NC score between each experimentally validated target was 0.5 or 1 (Figure 3B). The same tendencies were observed on other networks (Figure S1).

### Performance of mirTarPri

The above results indicated that that most of the gene groups targeted by the same miRNA had higher FS and NC score than random test groups. Therefore, the functional properties of these targets could be used for target analysis. For each individual functional genomics data (Gene Ontology and PPI network), to assess the ability of our approach in recognizing experimentally validated targets of corresponding miRNAs, we performed a large scale leave-one-out cross validation. In each validation run, one experimental validated target termed as ‘testing gene’, was deleted from training sets and added to 99 randomly selected genes (see Materials and Methods). mirTarPri then localized the rank positions of these testing genes for each functional genomics data. In validation tests of our study, if the testing miRNA-target interaction was involved in known interactions used by the algorithm, then current miRNA-target interaction was removed from the known validated miRNA-target dataset in this validation run. This procedure was applied to all following tests performed.

We calculated sensitivity (frequency of testing genes that were ranked above a particular cut-off point) and specificity (the percentage of genes ranked below the cut-off point) for these rank positions. We plotted receiver operating characteristic (ROC) curves considering the functional properties of the targets to facilitate the comparison between different functional genomics data. In a ROC curve, the sensitivity (true positive rate) is plotted in function of the 1-specificity (false positive rate) for different threshold. The AUC score is the most frequently used measure to evaluate algorithm performance. For example, an AUC score of 1 suggests that every testing gene ranked prior to other genes whereas a value of 0.5 indicates that the testing genes were randomly ranked along the list.

**Figure 4:**
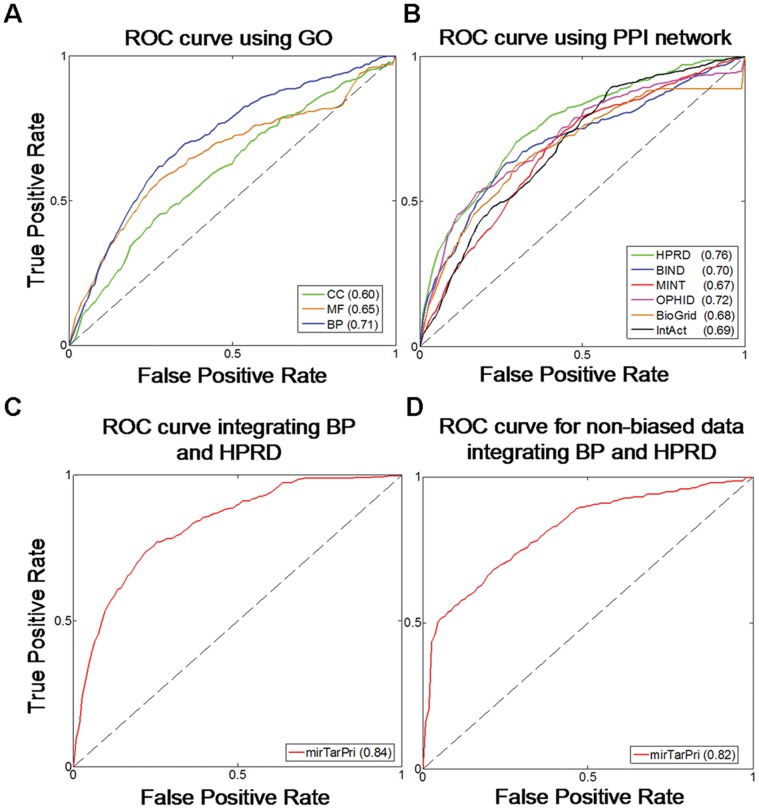
Cross validation results. (A) ROC curves based on the three orthogonal ontologies of GO. The maximum AUC score was 0.71 when using BP. (B) ROC curves based on six PPI networks. The maximum AUC score was 0.76 when using HPRD. (C) ROC curve of mirTarPri integrated BP and HPRD. After integrating multiple functional genomics data, the maximum AUC score of mirTarPri reached 0.84, which exceeded the AUC scores using a single dataset. (D) ROC curve of mirTarPri in testing unbiased targets identified by microarray or pSILAC from human normal cells.

For each functional genomics data source, mirTarPri reached an AUC higher than 0.5, indicating that it was a sensitive and specific means of ranking potential targets regardless of the data source that was used (Figure 4A, Figure 4B, Figure S2A and Figure S2B). Although the tested genes tended to rank highly in the priority list, this was not always the case. To minimize variability and increase ranking performance, mirTarPri integrated the BP (blue curve in Figure 4A) and HPRD (green curve in Figure 4B) ranks, which performed better than other AUC scores in their functional context. A final rank was generated using the Q statistic method (see Materials and Methods). This integrated rank performed better than all other ranks and yielded the highest AUC score (0.84). The AUC scores obtained using Gene Ontology and human PPI network were 0.71 and 0.76, respectively, compared with 0.49 for the randomly selected genes. The integrated rank yielded the highest AUC of 0.84 (red curve in Figure 4C and Figure S2C). In addition, to determine whether it was possible to use the same approach on data from other organisms, we tested our validation method on Arabidopsis thaliana by integrating BP ontology and MINT data. In total, 69 experimentally validated miRNA-target pairs were collected. Our method achieved an AUC score of 0.90 and high precision in cross validation (Figure S3).

Based on performance of mirTarPri in the context of multiple functional genomics data, we used the BP-HPRD integrated strategy for mirTarPri in experiment described below. Users of the mirTarPri online software can choose multiple combinations of all functional genomics data for different purposes.

### mirTarPri is an Unbiased Method

It is well known that many of validated targets are involved in cancer development. To test whether mirTarPri is capable to rank non-cancer related targets, validation was performed with high-throughput evidence obtained by microarray and pulse-labeing SILAC (pSILAC) technique which was used in other miRNA targets prediction validation works [12], [36], [57], [58]. 727 miRNA-target pairs identified by microarray or pSILAC from human normal cells were tested in this step. Each test gene was added into 99 randomly selected genes and prioritized by mirTarPri. ROC curve for validating these non-cancer-related targets was generated with AUC score up to 0.82 (red curve in Figure 4D and Figure S2D), slightly lower than 0.84 (red curve in Figure 4C). This result indicated that mirTarPri was an unbiased method.

### Prioritization of Existing Target Prediction Databases

To test the efficacy and precision of mirTarPri in predicting miRNA targets, we compared mirTarPri with six commonly used databases (TargetScan, PicTar, miRanda, PITA, DIANA-microT and RNAhybrid) to demonstrate the improvements gained from the multiple functional genomic data sets. If our method is successful in improving target prediction, then the experimentally validated targets will tend to be localized at the top of prioritized lists based on functional similarities to the training genes used for the corresponding miRNAs.

**Figure 5:**
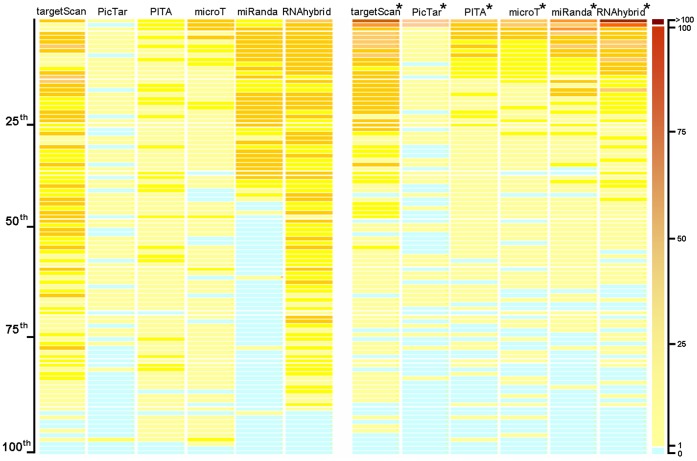
The performance of mirTarPri in prioritizing previously validated miRNA targets. A comparison of lists provided by six miRNA target prediction databases (TargetScan, PicTar, PITA, microT, miRanda and RNAhybrid) was made to lists prioritized by mirTarPri. The left panel displays the rankings produced by six miRNA target prediction tools for a set of validated gold standard targets used as test cases (orange lines), where the right panel displays the rankings after prioritization by mirTarPri (*). The distribution of validated target positions after prioritization by mirTarPri was shifted toward the top 20^th^ percentile compared with those prioritized by the other programs. All data are displayed in percentiles. The position line is a deeper orange color when more than one validated target is placed in this position.

Rigorous evaluations of a prediction method require gold standard data. In this step, a compendium of 1,556 miRNA-target pairs that were supported by strong experimental evidence (reporter assay or western blot analysis) was downloaded from mirTarBase (release 2.5) [59]. There were 560 overlapping cases among the 1,556 pairs and 1,799 cases derived from TarBase, miRecord and miR2Disease. The remaining 996 miRNA-target pairs were used as gold standard data (Table S1), which were predicted by each of the six methods tested in the present study. Next, we prioritized the target lists using mirTarPri and mapped the gold standard targets to the original lists from the six databases and prioritized mirTarPri lists for corresponding miRNAs (Figure 5).

Of the 996 validated target genes, TargetScan, PicTar, PITA, DIANA-microT, miRanda and RNAhybrid predicted 801, 255, 366, 294, 472 and 933, respectively. These predictions were uniformly distributed along the ranked lists from each method. In contrast, the same genes were preferentially distributed at the top of the prioritized rank lists produced by mirTarPri (Figure 5). We found that 111 of 801 (13.86%), 48 of 255 (18.82%), 54 of 366 (14.75%), 83 of 294 (28.23%), 121 of 472 (25.64%) and 175 of 933 (18.76%) validated targets fell within the top 10^th^ percentile in the original TargetScan, PicTar, PITA, DIANA-microT, miRanda and RNAhybrid lists, respectively. In particular, we found that 374 of 801 (46.69%), 92 of 255 (36.08%), 149 of 366 (40.71%), 137 of 294 (46.60%), 223 of 472 (47.25%) and 434 of 933 (46.52%) validated targets fell within the top 10^th^ percentile of the mirTarPri prioritized lists. The average numbers of predicted targets present in each list were comparable and ranged from 700 to 20,000 (see the mirTarPri website).

To quantify these results, we calculated the mean ES value of the 996 gold standard targets based on the original ranks produced by each target prediction method and the new ranks by mirTarPri (Figure S4). After prioritization, the mean ES values for these target genes were significantly increased: TargetScan, from 27.09 to 86.13 (3.18-fold); PicTar, 10.52 to 18.80 (1.79-fold); PITA, 13.66 to 34.75 (2.54-fold); DIANA-microT, 17.97 to 31.03 (1.73-fold); miRanda, 22.49 to 52.50 (2.33-fold); and RNAhybrid, 45.60 to 110.73 (2.43-fold). Thus, mirTarPri performed better than these traditional miRNA target prediction methods.

### Comparison with Other Integrated Methods

**Figure 6:**
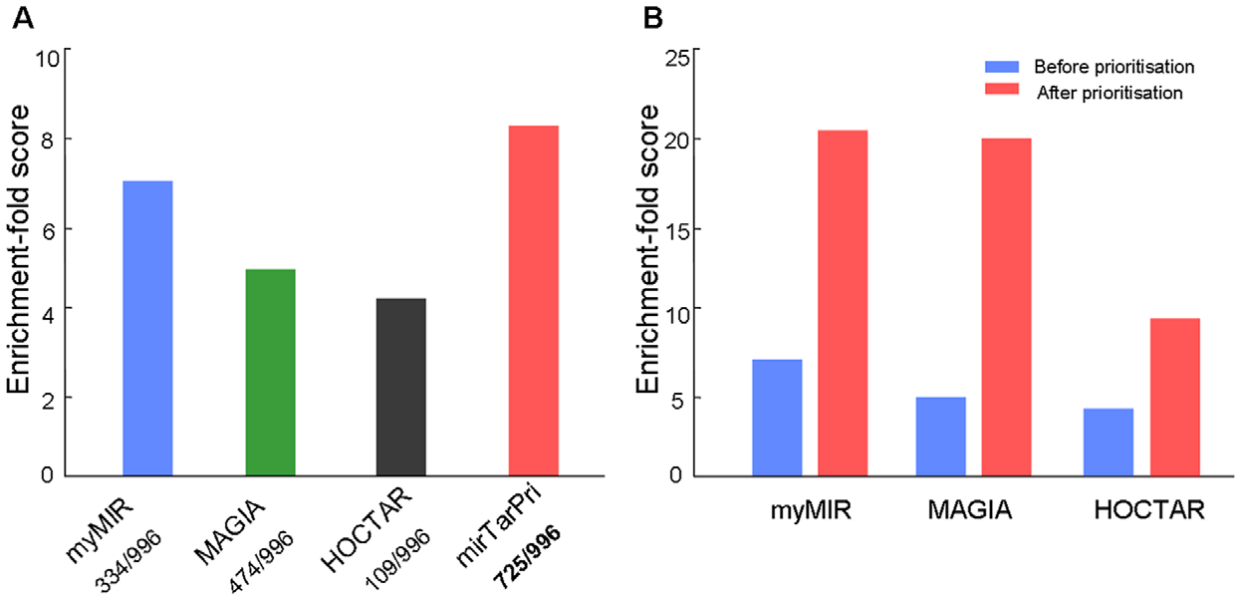
Comparison with other integrated methods. (A) mirTarPri recognized the most targets of golden standard data and got the highest ES of 8.25. (B) After prioritization of mirTarPri, ES of three methods were greatly increased.

Furthermore, mirTarPri was compared with other integrated methods, such as myMIR, MAGIA and HOCTAR [15], [58], [60]. myMIR collected and filtered predictions from TargetScan, miRanda, PicTar and DIANA-microT using the target accessibility feature from PITA. MAGIA allowed Boolean combinations to be retrieved from TargetScan, miRanda and PITA and integrated miRNA-mRNA expression. HOCTAR ranked the predictions of miRanda, TargetScan and PicTar on the basis of their anti-correlated expression behavior relative to their respective miRNA host genes. mirTarPri lists were generated by pooling the prioritized miRanda, TargetScan and PicTar results. 996 miRNA-target pairs (gold standard data) were mapped to lists for each method being compared and evaluated by ES. myMIR and MAGIA predicted 334 and 474, respectively, out of the total number of 996 strongly experimentally validated targets. HOCTAR predicted only 109 of these targets because it is based on the analysis of expression correlations between host genes and the targets of the corresponding intragenic miRNAs, but most miRNAs are non-intragenic and have no host genes. In comparison, mirTarPri predicted 725 out of the 996 strongly experimentally validated targets and had the highest mean ES (Figure 6A) and AUC score (Figure S5). These observations indicated that mirTarPri performed better than other integrated systems at recognizing and prioritizing miRNA target lists.

**Figure 7:**
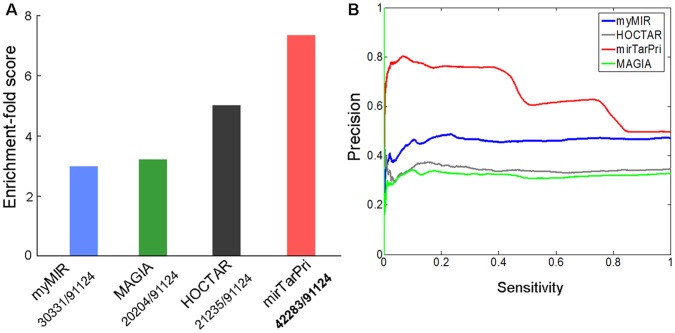
Comparison with other methods based on PAR-CLIP data. (A) mirTarPri recognized the most targets and had the highest ES of 7.34. (B) Curves showing prediction precision versus sensitivity indicated that mirTarPri performed better than other methods.

To further assess the performance of mirTarPri, we used data generated by PAR-CLIP (Photoactivatable-Ribonucleoside-Enhanced Crosslinking and Immunoprecipitation), an improved cross linking approach for directly identifying transcriptome-wide mRNA-binding sites for regulatory miRNA-containing ribonucleoprotein complexes [61] and HITS-CLIP (high-throughput sequencing of RNAs isolated by cross linking immunoprecipitation), a method to covalently crosslink native Argonaute protein-RNA complexes in mouse brain [62]. These data were collected and compiled by starBase [63]. In comparison with the other methods, mirTarPri recognized the greatest number of 91,124 cases and had the highest prioritizing ES and precision (Figure 7). We next divided the targets prioritized by mirTarPri into four groups according to the number of miRNA binding sites that they contained (1, 2, 3 or ≥ = 4). We found that targets with multiple binding sites tended to be more highly prioritized by mirTarPri (Figure S6). mirTarPri also performed well on mouse HITS-CLIP data in recognizing and prioritizing 98,517 cases (Figure S7).

### mirTarPri Online

We developed a web-based server for implementing the mirTarPri method (Figure S9), which is supported by a Tomcat 6.0 sever and MYSQL 5.5 database. mirTarPri online is freely accessible for non-commercial use at http://bioinfo.hrbmu.edu.cn/mirTarPri (or http://210.46.85.180∶8080/mirTarPri). The mirTarPri working principles and users’ manual can be accessed on the HELP page. mirTarPri provides two programs: (1) Prioritizing inputted candidate target lists according to a miRNA. Users can choose combinations of multiple functional genomic data sets for different purposes; and (2) Searching for mirTarPri prioritized miRNA target lists based on six prediction methods. mirTarPri also allows downloading of corresponding datasets and includes links to relative web sites.

## Discussion

In this present study, we showed that target genes of the same miRNA tend to share similar functional categories and tend to cluster together in the PPI network. Taking advantage of these functional similarities, we developed a method named mirTarPri to integrate functional information and prioritized miRNA target gene lists provided by commonly used target prediction databases, and demonstrated that mirTarPri was a valuable tool for improving of miRNA target prediction.

We extensively validated our method in a large-scale leave-one-out cross validation study using 1,799 validated miRNA-target pairs. For every functional data source, mirTarPri displayed higher AUC for predicted target genes than randomly selected genes. After integration, the BP-HPRD integrated rank provided by mirTarPri yielded the highest AUC of 0.84, indicating specific and sensitive in ranking candidate genes. Applying mirTarPri to high-throughput data indicated that mirTarPri was capable to rank both cancer and non-cancer related targets. For target prediction, we used mirTarPri to prioritize the results of six commonly used and well-established miRNA target prediction databases (TargetScan, PicTar, PITA, DIANA-microT, miRanda and RNAhybrid), which have previously been shown to be effective. We evaluated the efficiency of our procedure by analyzing a set of 996 previously validated miRNA-target pairs. Thus, the prioritized results represented a remarkable improvement. Comparison with other integrated systems indicated that mirTarPri performed better than other integrated systems in recognizing and prioritizing miRNA target lists. mirTarPri was also a flexible way to rank other target prediction methods.

For miRNA target prediction, most efforts have concentrated on the identification of seed-matching pairs. However, some validated miRNA target sites do not contain a complete seed match, indicating that perfect seed pairing is not a reliable criterion for predicting miRNA-target interactions [14]. Understanding the regulatory mechanisms of miRNAs in functional categories and complex interactions is essential for the discovery of functional miRNA-target pairs in complex cellular systems. mirTarPri can compensate for the limitations of seed-matching models. To the best of our knowledge, mirTarPri is the first tool to prioritize candidate miRNA target lists by systematically integrating multiple sources of functional genomics data. Therefore, mirTarPri is a novel tool for predicting miRNA targets.

In our previous study, we performed a framework to prioritize cancer risk miRNAs in a similar way used Gene Ontology data only [37]. Although achieved remarkable success, it overlooked the contribution from other functional data sets for studying gene sets association. In this work, we fused multiple functional data sets and used Q statistic method to integrate separate functional correlation prioritization ranks into a single rank. This strategy can handle missing annotated genes and minimize bias for well-annotated targets. We also used fold-enrichment measurements to convert the performance of mirTarPri in prioritizing candidate gene lists into a quantifiable score.

There were 94 miRNAs that had a single experimentally validated target in our collection after combining data from TarBase, miR2Disease and miRecord. Because leave-one-out cross validation can only be carried out with more than two targets, these single-target miRNAs were not included in the leave-one-out cross validation process. Apart from this, all miRNAs were included in the following prioritization and comparison. Based on the single-target miRNAs, mirTarPri also successfully prioritized gold standard and CLIP targets (Figure S8).

mirTarPri prioritized existing miRNA target predictions based on multiple functional genomic data sets. Although no novel targets will be found in prioritized target lists, mirTarPri performed better than myMIR, MAGIA, and HOCTAR at recognizing positive targets and reducing the false-positive rates in the upper ranks. Currently, our method is suitable for prioritizing candidate targets for miRNAs with known targets. Fortunately, the number of experimentally validated miRNA targets has increased rapidly in recent years and many have known targets [59]. Therefore, with the continued growth of known miRNA target data, our method will be increasingly useful in future studies. We believe that mirTarPri will play an important role as a pre-processing step to guide ‘wet’ lab experimental designs.

In conclusion, we presented a computational method for efficiently prioritizing miRNA candidate target lists. We believe that our method will significantly contribute to the fast-growing number of publicly available functional data sources and to the development of comprehensive biological categories for functional characterization.

## Supporting Information

Figure S1 **An illustration of the functional similarity between genes targeted by hsa-miR-7.** Five target genes (PAK1, SNCA, EGFR, IRS1 and IRS2) were mapped GO: BP (A), MF (B), CC (C) and PPI network: HPRD (D), BIND (E), BioGrid (F), IntAct (G), MINT (H),OPHID (I). Four targets were mapped to common terms GO: 0005634 and GO: 0005829 in BP (A), The same trends were observed for the MF and CC. Five target gene products (light blue) were close to each other on the HPRD network, and the average shortest distance was 1.70 (D). The same trends were observed for other networks: the target genes in PPI networks occupied a narrow niche.
(TIF)

Figure S2 **Curves showing prediction precision versus sensitivity was also generated for leave-one-out cross validation.** (A) Analysis based on the three orthogonal ontologies of GO. BP performed better than other ontologies with high precision. (B) Analysis base on six PPI networks. HPRD performed better than other networks with high precision. (C) pROC curve of mirTarPri integrated BP and HPRD. (D) pROC curve of mirTarPri in testing unbiased targets identified by microarray or pSILAC from human normal cells.
(TIF)

Figure S3 **Leave-one-out cross validation results for 69 Arabidopsis thaliana genes based on integrated BP ontology and MINT data.** (A) ROC curve for the validation with an AUC of 0.90. (B) pROC curve for the validation showing high prediction precision versus sensitivity.
(TIF)

Figure S4 **Quantity comparison of mirTarPri (red) with six target predictions in prioritizing previously validated miRNA targets (blue).** The values were calculated using the enrichment-fold method.
(TIF)

Figure S5 (A) ROC curves for mirTarPri in comparison with three other methods using 996 gold standard data. (C), (E), (G) After prioritization of mirTarPri (*), AUC scores of three methods were greatly increased than Before prioritization. (B), (D), (F), (H) Corresponding curves showing prediction precision versus sensitivity. The precision of these three methods was increased after prioritization (*).
(TIF)

Figure S6 **PAR-CLIP identified targets were categorized according to the miRNA binding number they contained.** Targets with multiple binding sites tended to be prioritized forward by mirTarPri.
(TIF)

Figure S7 **Comparison with other methods based on mouse HITS-CLIP data.** (A) mirTarPri recognised the most targets and had the highest ES of 5.96. (B) Curves showing prediction precision versus sensitivity indicated that mirTarPri performed better than other methods. For HOCTAR, MAGIA and myMIR only consider on human miRNA target prediction, mirTarPri was not compared with these methods for mouse HITS-CLIP data.
(TIF)

Figure S8 **Based on single-target miRNAs, mirTarPri successfully prioritized gold standard and PAR-CLIP targets.** For the gold standard targets, mirTarPri had the highest ES of 9.40 (A) and the highest precision (B). For the PAR-CLIP data, mirTarPri had the highest ES of 6.79 (C) and highest precision (D).
(TIF)

Figure S9 **An overview of the mirTarPri online framework.** (1) Prioritize user input candidate target list based on multiple functional genomics data; (2) Search mirTarPri prioritized miRNA target prediction databases; (3) Download corresponding data sets; (4) Links to relative functional data sources; and (5) Downloadable description of the mirTarPri working principle and users’ manual.
(TIF)

Table S1 **Detailed information for 996 gold standard targets tested by mirTarPri.**
(XLS)
