# Supplementary figures and images for: mirTarPri: Improved Prioritization of MicroRNA Targets through Incorporation of Functional Genomics Data
Source: PLoS One. 2013 Jan 9;8(1):e53685. doi: 10.1371/journal.pone.0053685 (PMC3541237; doi:10.1371/journal.pone.0053685)

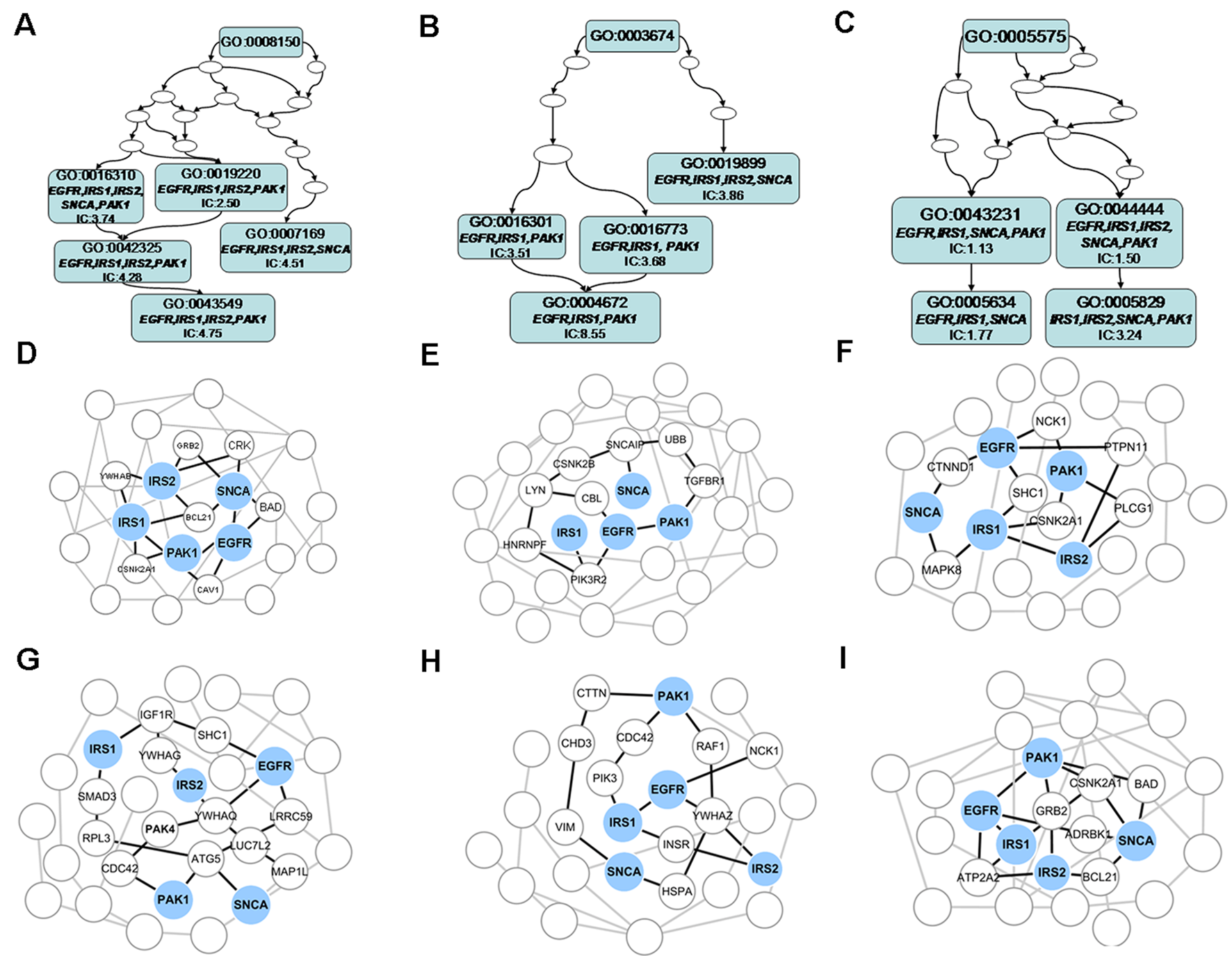

Supplement: Figure S1 — An illustration of the functional similarity between genes targeted by hsa-miR-7. Five target genes (PAK1, SNCA, EGFR, IRS1 and IRS2) were mapped GO: BP (A), MF (B), CC (C) and PPI network: HPRD (D), BIND (E), BioGrid (F), IntAct (G), MINT (H),OPHID (I). Four targets were mapped to common terms GO: 0005634 and GO: 0005829 in BP (A), The same trends were observed for the MF and CC. Five target gene products (light blue) were close to each other on the HPRD network, and the average shortest distance was 1.70 (D). The same trends were observed for other networks: the target genes in PPI networks occupied a narrow niche. (TIF) [file pone.0053685.s001.tif]

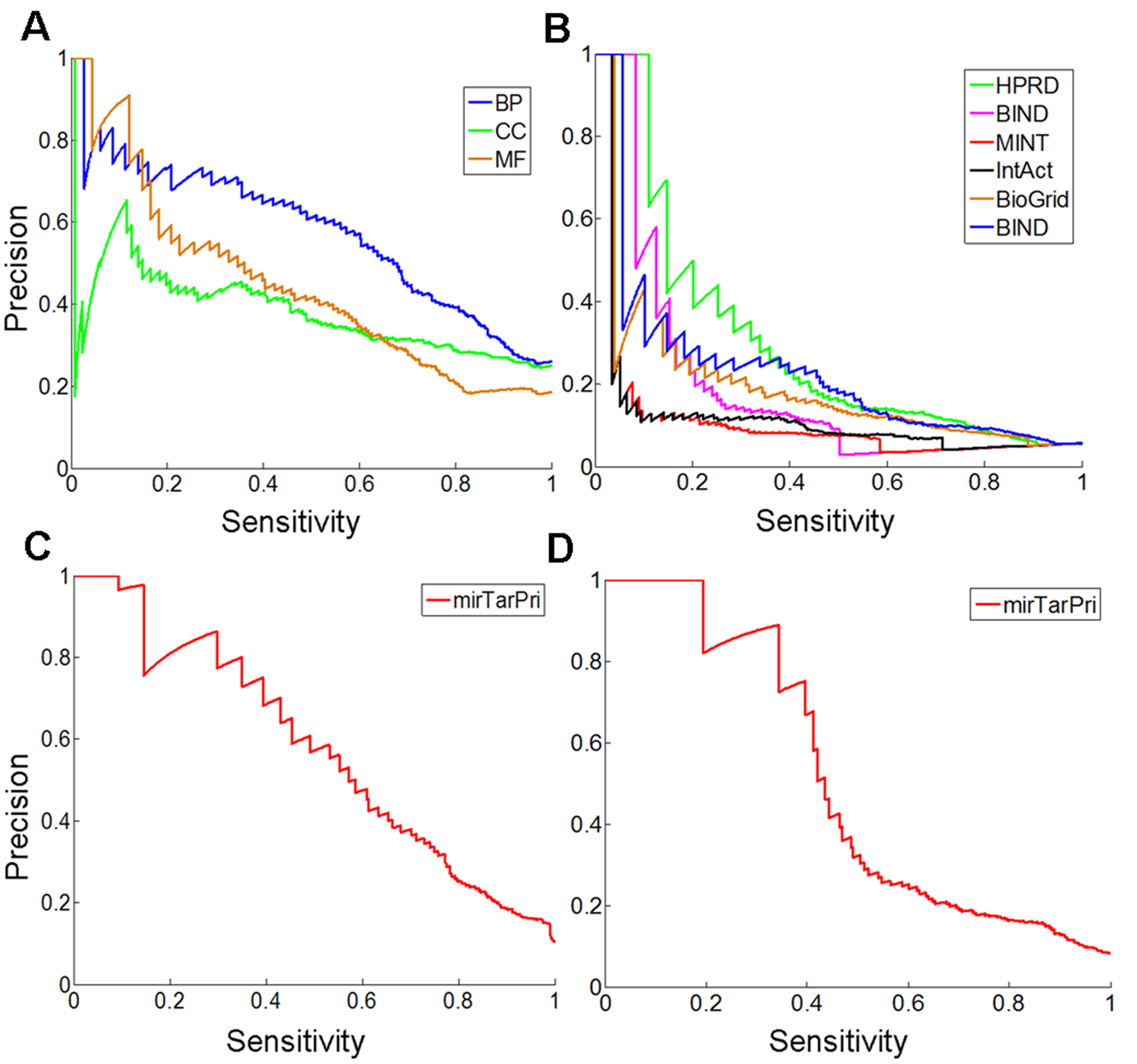

Supplement: Figure S2 — Curves showing prediction precision versus sensitivity was also generated for leave-one-out cross validation. (A) Analysis based on the three orthogonal ontologies of GO. BP performed better than other ontologies with high precision. (B) Analysis base on six PPI networks. HPRD performed better than other networks with high precision. (C) pROC curve of mirTarPri integrated BP and HPRD. (D) pROC curve of mirTarPri in testing unbiased targets identified by microarray or pSILAC from human normal cells. (TIF) [file pone.0053685.s002.tif]

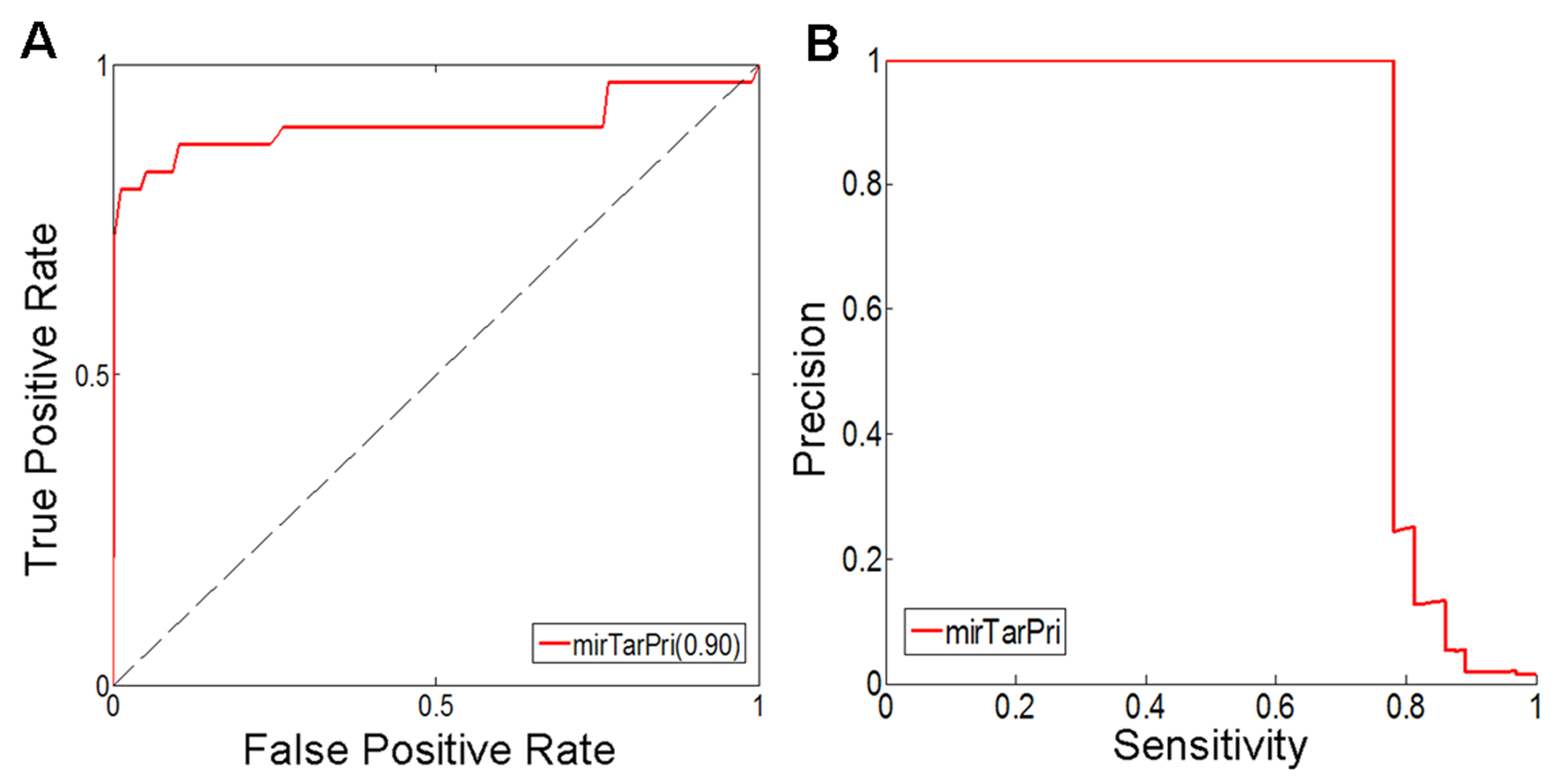

Supplement: Figure S3 — Leave-one-out cross validation results for 69 Arabidopsis thaliana genes based on integrated BP ontology and MINT data. (A) ROC curve for the validation with an AUC of 0.90. (B) pROC curve for the validation showing high prediction precision versus sensitivity. (TIF) [file pone.0053685.s003.tif]

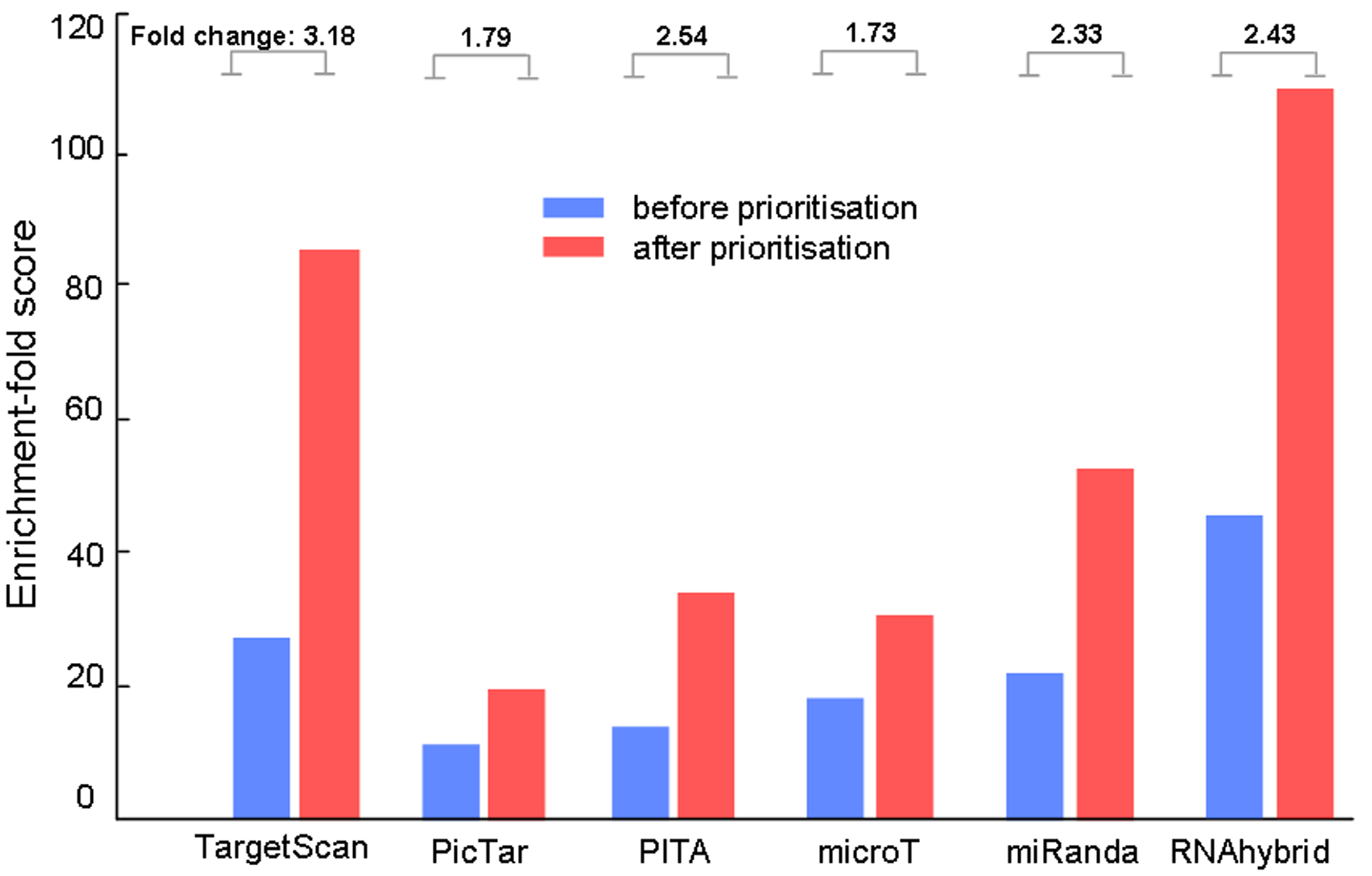

Supplement: Figure S4 — Quantity comparison of mirTarPri (red) with six target predictions in prioritizing previously validated miRNA targets (blue). The values were calculated using the enrichment-fold method. (TIF) [file pone.0053685.s004.tif]

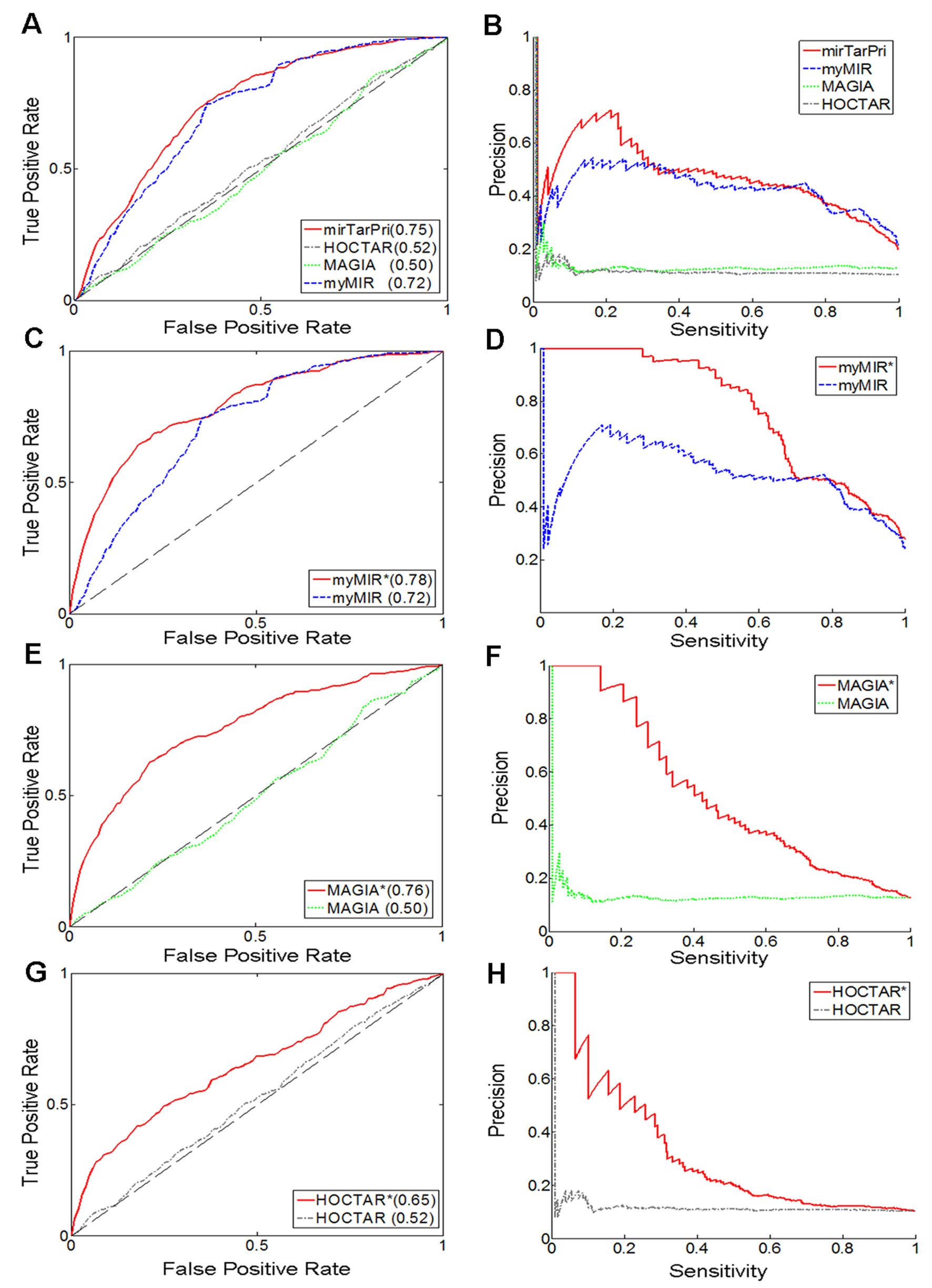

Supplement: Figure S5 — (A) ROC curves for mirTarPri in comparison with three other methods using 996 gold standard data. (C), (E), (G) After prioritization of mirTarPri (*), AUC scores of three methods were greatly increased than Before prioritization. (B), (D), (F), (H) Corresponding curves showing prediction precision versus sensitivity. The precision of these three methods was increased after prioritization (*). (TIF) [file pone.0053685.s005.tif]

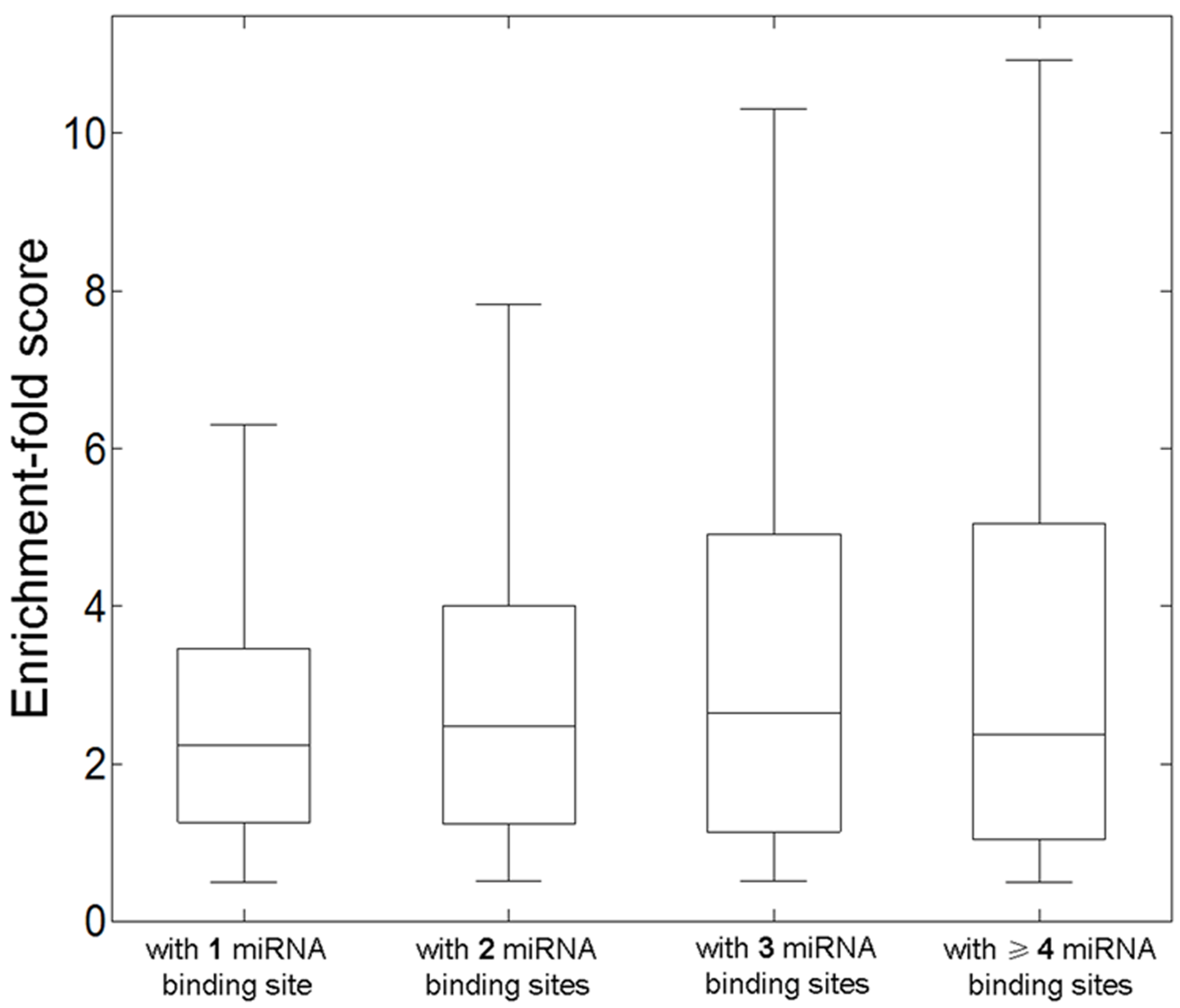

Supplement: Figure S6 — PAR-CLIP identified targets were categorized according to the miRNA binding number they contained. Targets with multiple binding sites tended to be prioritized forward by mirTarPri. (TIF) [file pone.0053685.s006.tif]

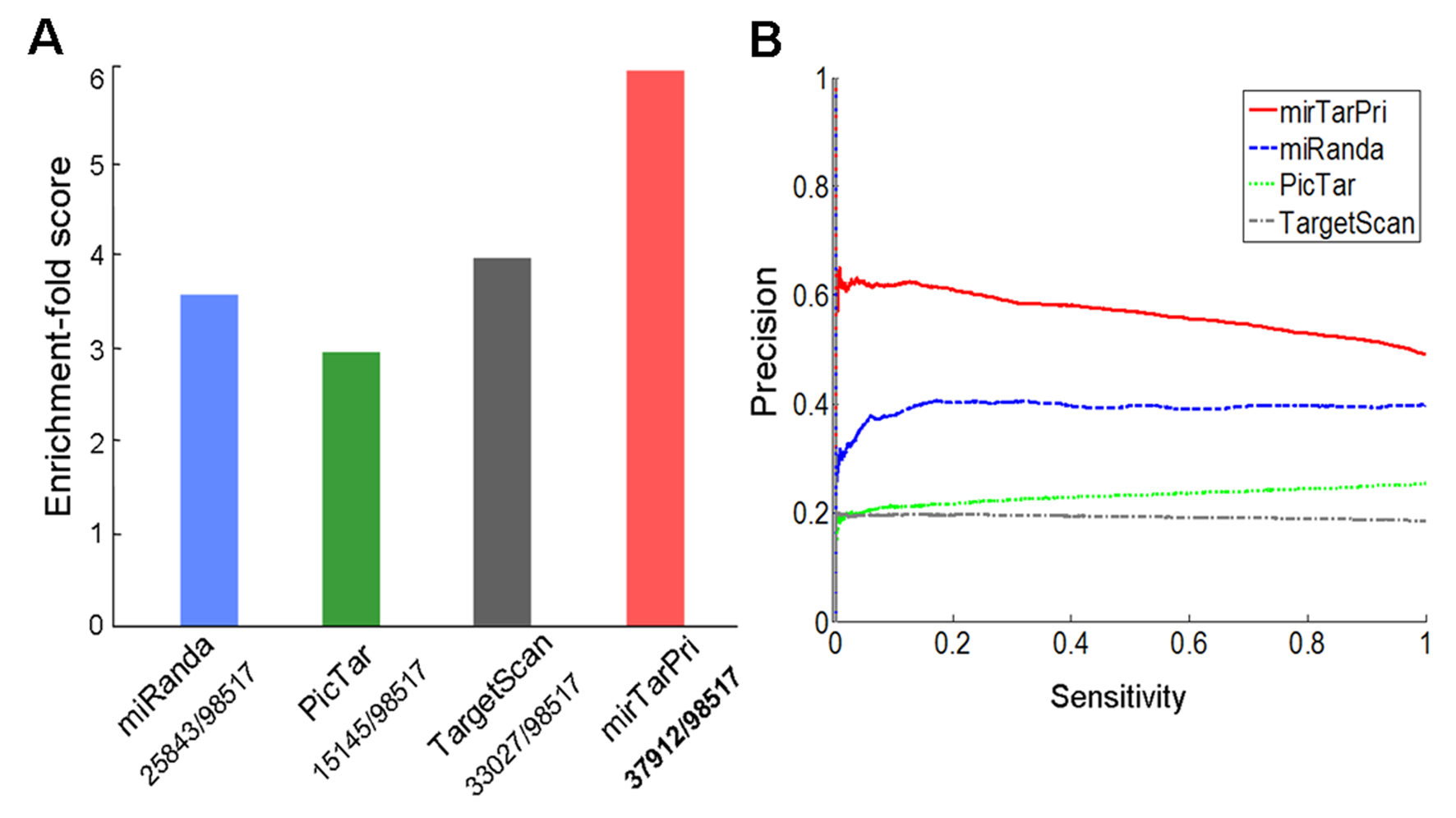

Supplement: Figure S7 — Comparison with other methods based on mouse HITS-CLIP data. (A) mirTarPri recognised the most targets and had the highest ES of 5.96. (B) Curves showing prediction precision versus sensitivity indicated that mirTarPri performed better than other methods. For HOCTAR, MAGIA and myMIR only consider on human miRNA target prediction, mirTarPri was not compared with these methods for mouse HITS-CLIP data. (TIF) [file pone.0053685.s007.tif]

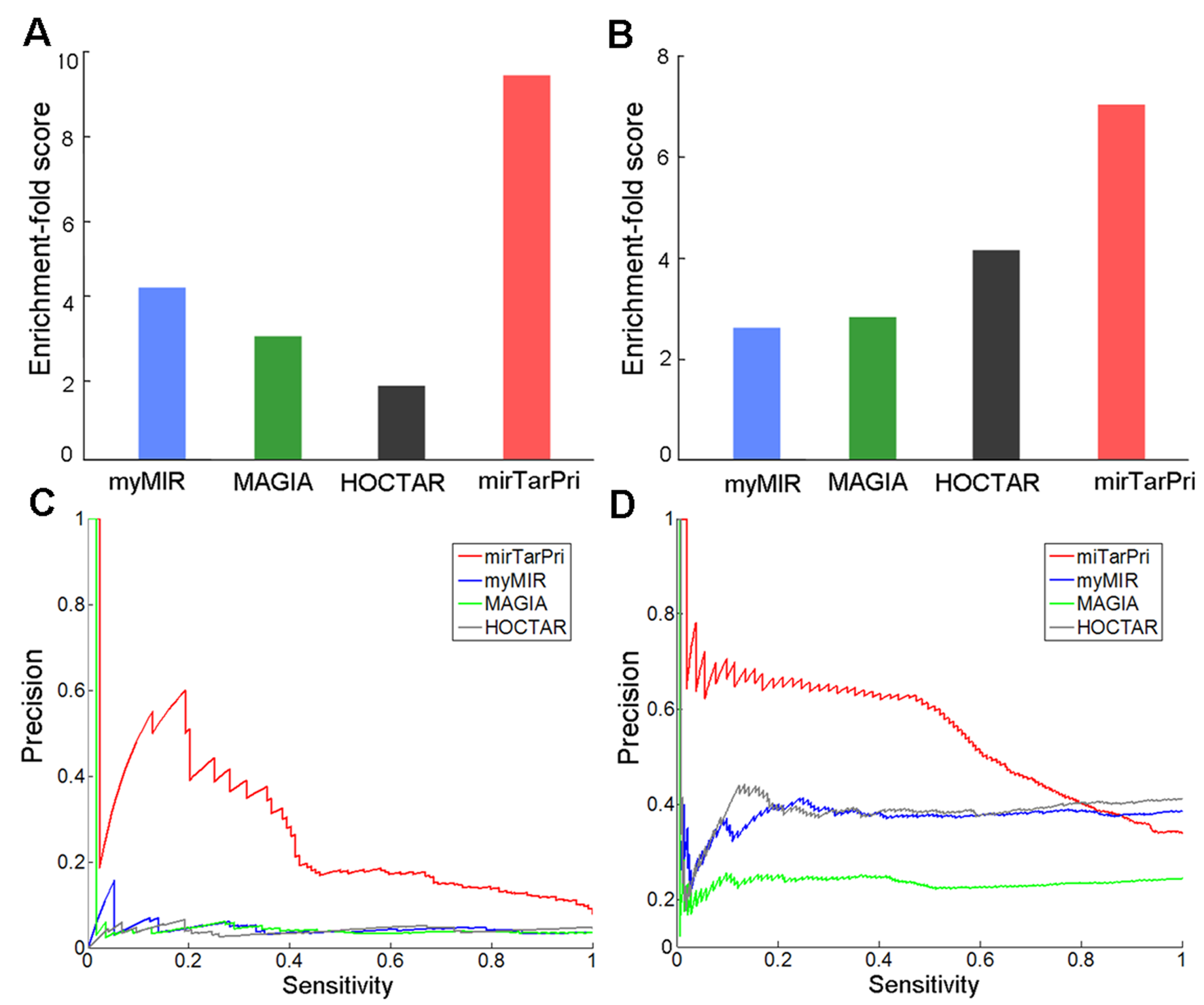

Supplement: Figure S8 — Based on single-target miRNAs, mirTarPri successfully prioritized gold standard and PAR-CLIP targets. For the gold standard targets, mirTarPri had the highest ES of 9.40 (A) and the highest precision (B). For the PAR-CLIP data, mirTarPri had the highest ES of 6.79 (C) and highest precision (D). (TIF) [file pone.0053685.s008.tif]

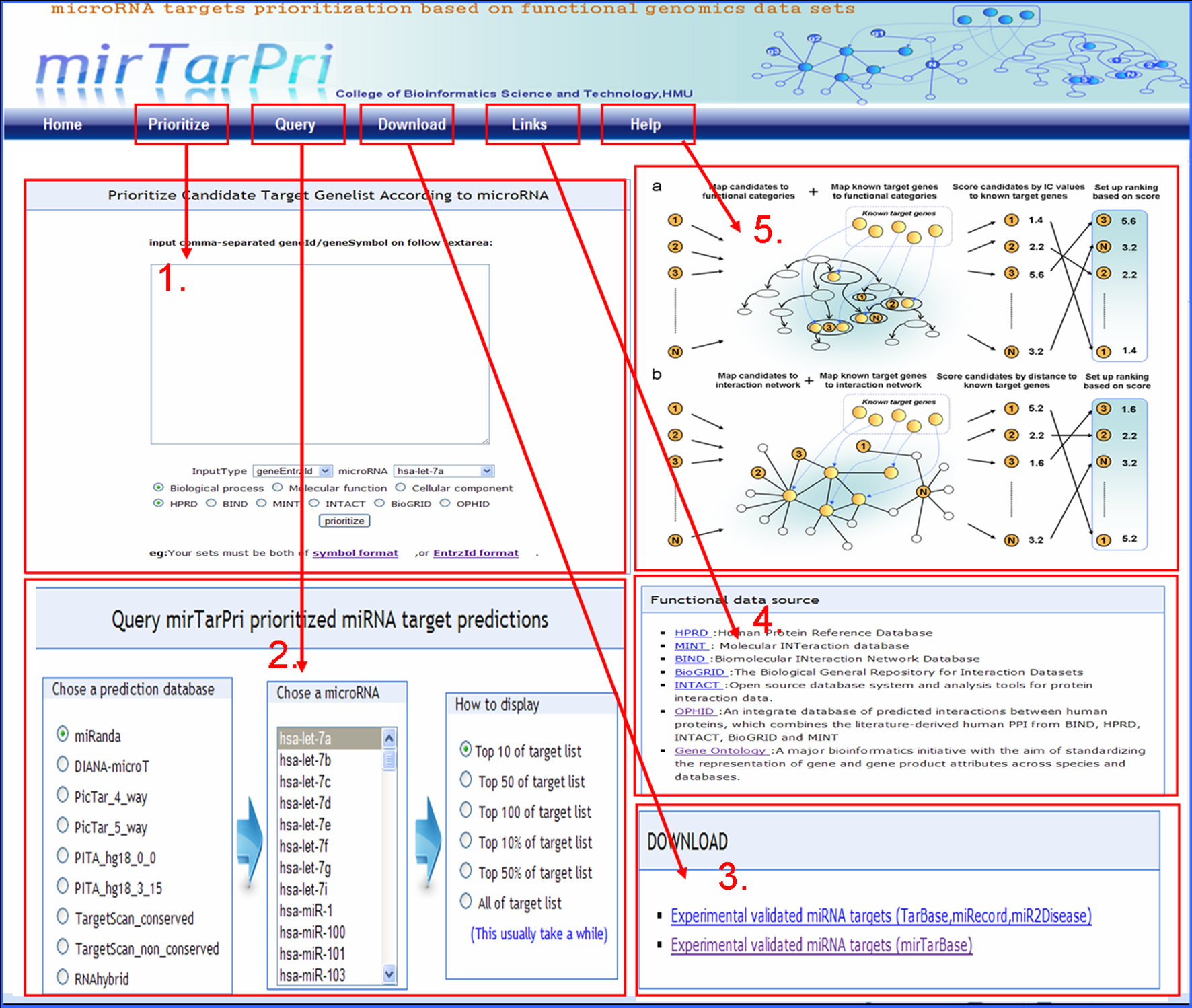

Supplement: Figure S9 — An overview of the mirTarPri online framework. (1) Prioritize user input candidate target list based on multiple functional genomics data; (2) Search mirTarPri prioritized miRNA target prediction databases; (3) Download corresponding data sets; (4) Links to relative functional data sources; and (5) Downloadable description of the mirTarPri working principle and users’ manual. (TIF) [file pone.0053685.s009.tif]
